# Philadelphia Academy of Surgery

**Published:** 1894-01

**Authors:** 


### Society Reports.

### PHILADELPHIA ACADEMY OF SURGERY.

MEETING OCTOBER 2, 1893.

The President, DR. WILLIAM HUNT, in the chair.

DR. OSCAR H. ALLIS rėad a paper on

### CARBOLIC ACID USED IN FULL STRENGTH IN SURGERY.

(See page 11.)

DISCUSSION.

DR. H. R. WHARTON: I would like to ask if Dr. Allis has seen carbolic acid poisoning from the use of the agent in this way. I have never seen much trouble from the use of the carbolic acid except in children. At the Children's Hospital I have seen two or three cases where its use has produced a marked constitutional effect. In one instance where a large nævus was dressed with carbolic acid application there was a dark-colored urine and other symptoms of poisoning.

DR. WILLIAM J. TAYLOR: I think the application of pure carbolic acid to a fresh, clean surface, such as is left after the removal of the breast, is totally unnecessary. If you have a thoroughly clean skin, clean instruments, ligatures, and hands, you will have primary union. If such a fresh surface is smeared with carbolic acid there will be a large amount of oozing. My experience with a few cases where strong carbolic acid solutions were used a number of years ago was that healing was much retarded.

As an application to suppurating surfaces such as Dr. Allis speaks of, and where you wish a cauterizing and disinfecting action, I consider carbolic acid one of the best agents that we have, and use it frequently.

DR. W. JOSEPH HEARN: If carbolic acid is applied to a raw surface, otherwise healthy, one would expect to have a certain amount of necrosis of the tissues. Some cells will be destroyed, and afford a soil for the propagations of germs.

DR. RICHARD H. HARTE: Dr. Levis was in the habit of using carbolic acid for its cautery effect. I remember several cases where he used it freely, producing large sloughs over the posterior surface of the thigh.

DR. ALLIS: In regard to poisoning Dr. Gardner claims immunity from poisoning from the fact that the application sears the whole surface and closes the small vessels, and nothing is taken into the system. Dilute solutions are rapidly taken up. In one case where I operated on two herniæ in the same individual there was a good deal of collapse following the use of a dilute solution of carbolic acid.

I am not prepared to say whether it has a necrotic action or not. I do not understand how Dr. Gardner gets pimary union using it as he does if it has such an action.

I think that Dr. Gardner probably began its use with the idea that there might be left after amputation of the breast some cells which it would destroy. I do not bring this forward thinking that anyone will be led to use it in these cases, but there is a big lesson in this use of the carbolic acid. There are places where it is valuable, for instance, in deep sinueses and pus tracts. I have injected it into a psoas abscess so that it would run out—probably eight ounces—without the slightest constitutional effect.

I can subscribe to what Dr. Harte says. Care must be taken that the carbolic acid does not come in contact with the skin, If it touches the skin it will blister it, but when applied to a raw surface it does not have the effect which we should expect. In a few cases where it has been injected into the tunica vaginalis the patients have almost died, but in a large majority of cases carbolic acid pure in hydrocele effects a happy cure and without suppuration. Hence without necrotic action.

In collecting some cases of accidents in the treatment of hydrocele such cases were reported to me.

As to whether or not the application in recent surgery is necessary or advantageous I leave that for individual opinion. I

have seen bichloride solution do as much mischief as carbolic acid probably could do in preventing primary union.

DR. L. W. STEINBACH: In speaking of the use of carbolic acid Dr. Levis has been referred to. A number of years ago I had the pleasure of assisting Dr. Levis in the removal of an ovarian cyst in private practice. At that time the spray was used. The assistant who had charge of the spray put the carbolic acid in the bottle and the water on top of it without mixing the two, so that a spray of pure carbolic acid was delivered into the wound and on to the operator's hands. The doctor's hands became so benumbed that he was unable to introduce the stitches. The woman, however, made an excellent recovery.

Of course every one knows the good success of Dr. Levis in the treatment of hydrocele with carbolic acid. He was careful that none got into the connective tissues or on the scrotum. I never saw an accident in any of his numerous cases.

MEETING DECEMBER 4, 1893.

The President, DR. WILLIAM HUNT, in the chair.

---

DR. JOHN B. DEAVER read a paper on

REMARKS UPON THE TREATMENT OF STRICTURE OF THE SIGMOID FLEXURE AND OF THE FIRST PORTION OF THE RECTUM,

(which will appear in a following number.)

---

### REPORT OF A CASE OF TETANUS.

---

BX WILLIAM G. PORTER, M. D.

---

Helmer E., aged twenty-three years; nativity, Sweeden; occupation, machinist; weight. 214 pounds; height, six feet two inches. Patient presented an excess of adipose tissue; skin and mucous membranes pale; had had pneumonia in 1892; was abdicted to the use of alcohol, especially beer.

He was brought to the Philadelphia Hospital on the fourth day of the disease at 1 A. M., November 11, 1893, with the history of having run a rusty nail in the sole of his foot, two weeks previously, while working in a cellar.

The wound gave no trouble, healing in a few days. Ten days after the injury, he noticed some pain upon swallowing. The next morning his jaws were stiff, by evening he could not open his mouth, his back was stiff and the seat of severe pain, which he described as feeling like a band of constriction, passing from the back around the abdomen and down into the legs. Upon admission the jaws were firmly locked, the masseters hard, risus sardonicus present. There was a moderate degree of continuous opisthotonus, the abdominal muscles were board-like, and when lifted from the stretcher to the bed he moved as if made of one rigid piece, there being no motion in any joint from his head to his feet. Paroxysmal spasms occurred every one to three minutes, increasing the contractions of all the muscles involved in tonic spasm.

His pulse was soft and rapid, heart-sounds weak, temperature, 99,° bowels had not moved for three days. An evacuation was secured by means of croton oil and a laxative enema. A little ether was given and a free and deep incision made where the punctured wound had been, it was thoroughly cleansd with bichloride (1:200) and a wet bichloride dressing applied. He was placed upon the combined bromides gr. xiv, with chloral gr. xv, every three hours. Nitro-glycerine gt. j, every fifteen minutes till three doses were given, then every hour till there was flushing of the face, then every two hours.

Urinalysis showed a high colored urine, specific gravity 1033, a decided ring of albumin, an excess of chlorides, and a few granular casts.

The full effect of the chloral was not secured till the third day after admission, when there was given by the bowel during the afternoon ʒij of chloral with ʒv of bromides. He now slept soundly, and during the sleep there was less arching of the back and some relaxation of masseters. The paroxysmal spasms, however, did not seem to be influenced by the chloral. Its use by the bowel was continued, giving it in such doses and at such intervals as seemed indicated.

A free perspiration marked the entire course of the disease. Within a few hours after admission the temperature became normal, and remained so till the seventh day, when it began a slow but continuous ascent.

Feeding was not difficult, the nozzle of the feeding cup was placed between his lips and the contents found their own way to his throat, frequently, however, causing repeated spasms of the pharyngeal muscles. For the first few days he was eager for milk, and five pints were given daily, later he did care for nourishment, but continually craved beer. Eggs were given in milk and with sherry wine.

During the seventh day of the disease there was much less pain in the back. There was considerable less spasm, patient expressed himself as feeling much better. His mind had been perfectly clear up to this time, but during the night there was a little delirium, and a slight tendency to pick at the bed clothes.

Eighth day; Lower jaw drops a little during sleep, head not thrown back, paroxysms frequent but not severe. During the night considerable of delirium and some twitching of muscles and carphologia.

Ninth day: All continuous spasms had disappeared, mouth open, and a guard was placed between the teeth to protect the tongue being bitten during the paroxysms, which were marked by prompt snapping of the jaws. At 5:30 P. M. he had a prolonged and severe spasm, after which there was great muscular relaxation; urine and feces were passed involuntarilly, respirations became very shallow, no radical pulse could be felt, and he became profoundly unconscious.

At noon his temperature had been 104°; at 5:30 it was 106°; at 6, 107°; at 6:30, 108°; at 7, 109.° At 7:20 the heart ceased beating. Shortly after the cessation of respiration, one-half hour later, the temperature of the body was 111.° Antitoxine: Upon the evening of the seventh day the administration of antitoxine was begun. It was deeply injected into the muscles of the thigh twice daily in ascending doses. The first dose was 20 centigrammes, the last dose 160 centigrammes.

This treatment appeared to have no influence upon the symptoms. There were no local effects at the points of injection. The temperature had begun to rise before the first dose was given.

A post-mortem examination was not secured by the hospital authorities, but was, however, secured after the removal of the body. The membranes of the brain and cord were found intensely hyperæmic; the brain œdematous; the ventricles and central canal of the cord were distended with fluid. The brain and cord are preserved for further examination.

Heart flabby and relaxed. The blood in the auricles and great vessels was dark and fluid. Nothing noteworthy found in other organs.

For these notes and her untiring care of the patient, I am indebted to my very efficient resident, Dr. Emma J. Lucas. And for the opportunity of using the anti-toxine to Drs. Wm. E. Hughes and Carter, who furnished the material and made the injections.

---

DISCUSSION.

DR. JOHN B. ROBERTS: I have been much interested in Dr. Porter's account of the unsuccessful use of antitoxine in acute tetanus. I am inclined to believe, from what I have read, that most of the cases that have recovered have been rather mild cases, and that the more severe cases have not shown any very brilliant result from the use of such treatment.

---

DR. THOMAS G. MORTON exhibited a patient, aged twenty-two months, on whom he had operated for

EQUINO VARUS WITH DISLOCATION OF THE ASTRAGALUS, where he had removed the astragalus from each foot.

DR. JOHN B. DEAVER exhibited a patient showing the result of operation for the

RADICAL CURE OF HERNIA BY BASSINI'S METHOD.

The operation was performed on October 12th on account of temporarily irreducible hernia and the inability to properly control the hernia by a truss. The operation has the advantage of not confining the patent to bed for a long time. It consists practically of making a new inguinal canal. The hernia is exposed and the spermatic cord separated from the sac. The hernia is reduced and the sac tied off close within the abdominal cavity and the redundant portion cut away. The internal oblique and transversalis muscles, with the rectus, are then stitched to Poupart's ligament, the cord placed over these muscles, and the skin and superficial fascia over the cord. He used buried sutures of silk worm-gut, which were allowed to remain.

DR. JAMES M. BARTON: On several occasions I have started to perform Halstead's or Bassini's operation, but when I have gotten to the ring I have found the hernia so old that there was practically no canal, one ring lying directly over the other, and as it seemed to me impossible to perform either of these operations I have performed the MacEwen operation. In my earlier MacEwen operations I cut off all but four or five inches of the sac, when it was very long, fearing there might be sloughing if I retained it all, but in the later operations I have used the entire sac, even when a foot long. I would ask Dr. Deaver if in these cases of large hernia, where the two rings practically correspond, he makes any attempt to do Halstead's or Bassini's operation.

DR. J. B. DEAVER: In answer to Dr. Barton, I would state that I have done very few of the Bassini operations, but in such cases as Dr. Barton mentions I have done Barker's operation without, however, obtaining a permanent radical cure. This operation consists principally in tying off the sac at the internal abdominal ring. As Dr. Barton has pointed out, I think there would be some difficulty in doing Bassini's operation.

